# Quadruple term gestation of quadri-chorionic quadri-amniotic pregnancy after artificial insemination: a case report

**DOI:** 10.1186/s12978-022-01400-2

**Published:** 2022-04-21

**Authors:** Mauricio Caballero-Reyes, Diana Medina-Rivera, César Alas-Pineda, Beatriz Mejía-Raudales, Kristhel Gaitán-Zambrano, Tesla Valle Rubí

**Affiliations:** 1 Instituto Hondureño de Seguridad Social, San Pedro Sula, Cortés Honduras; 2 Departamento de Epidemiología, Hospital Dr. Mario Catarino Rivas, San Pedro Sula, Cortés Honduras; 3 Facultad de Medicina y Cirugía, Universidad Católica de Honduras - Campus San Pedro y San Pablo, San Pedro Sula, Cortés Honduras

**Keywords:** Multiple pregnancy, Assisted reproductive techniques, Quadruplet, embarazo múltiple, técnicas de reproducción asistida, cuatrillizos

## Abstract

**Background:**

To solve infertility, modern science has promoted assisted reproduction techniques such as in vitro fertilization, ovulation induction, and artificial insemination. Quadruple-type multiple pregnancies occur in 1 of every 500,000 pregnancies, and it is estimated that 90% occur due to assisted reproductive techniques, which often lead to numerous complications.

**Case presentation:**

Here we present a case of a 33-year-old woman, who desired pregnancy, but had a history of primary infertility diagnosed by hysterosalpingography, and endometriosis, which was treated by fulguration and medical management. Concomitantly, the patient was anovulatory. To fulfill her wish, she underwent homologous artificial insemination, after treatment, she successfully conceived quadri-chorionic quadri-amniotic infants, who were born at 37.2 weeks, without perinatal or maternal complications.

**Conclusion:**

This paper presented the parameters of prenatal care, appropriate management approach, and successful resolution without maternal–fetal complications despite the inherent risks of this type of pregnancy.

## Plain language summary

Infertility is a common reproductive system problem; modern science has promoted assisted reproduction techniques as an alternative for these cases. High-order pregnancies it is estimated to occur in 90% of the cases due to assisted reproductive techniques, which often lead to numerous complications. A 33-year-old woman, who desired pregnancy, but had a history of infertility underwent artificial insemination, conceiving successfully quadruplets without any perinatal or maternal complications. Knowledge of maternal history and proper management of assisted reproductive techniques in addition to adequate prenatal follow-up are necessary for a successful pregnancy.

## Resumen

**Introducción:**

Para solucionar la infertilidad, la ciencia moderna ha promovido las técnicas de reproducción asistida, como la fecundación in vitro, la inducción de la ovulación y la inseminación artificial. Los embarazos múltiples de tipo cuádruple se producen en 1 de cada 500.000 embarazos, y se estima que el 90% ocurren debido a las técnicas de reproducción asistida, que a menudo conllevan numerosas complicaciones.

**Presentación del caso:**

Presentamos el caso de una mujer de 33 años, que deseaba un embarazo, pero tenía antecedentes de infertilidad primaria diagnosticada por histerosalpingografía, y endometriosis, que fue tratada mediante fulguración y manejo médico. Al mismo tiempo, la paciente era anovulatoria. Para cumplir su deseo, se sometió a una inseminación artificial homóloga y, tras el tratamiento, concibió con éxito niños cuadri-coriónicos cuadri-amnióticos, que nacieron a las 37,2 semanas, sin complicaciones perinatales ni maternas.

**Conclusión:**

Este trabajo presentó los parámetros de atención prenatal, el enfoque de manejo adecuado y la resolución exitosa sin complicaciones materno-fetales a pesar de los riesgos inherentes a este tipo de embarazo.

## Background

According to the World Health Organization, one in four couples in developing countries is affected by infertility [1]. Infertility is defined as a disease of the reproductive system characterized by the inability to achieve a clinical pregnancy after 12 months of regular unprotected sexual intercourse. This concept aligns with expert consensus published in 2009; since then, it has not been modified [2]. Faced with this problem, modern science seeks to provide innovative solutions through assisted reproductive technologies (ART), which can be subdivided into two large well-differentiated groups: those with highly complex in vitro fertilization (IVF), and those with low- complexity ovulation induction and artificial insemination [3]. Triple, quadruple, or more fetuses (known as “high- order gestation”) are frequent results of ART and a greater use of ovulation inducers, making them responsible for 10–20% of pregnancies in infertility cases [4].

History shows the vast fascination that multiple pregnancies have generated due to their low incidence and limited documentation. The first report of quadruple pregnancies dates back to 1750, with the Smiths; however, in 1880, data were first obtained as first quadruplets reached adulthood. Over the years, the scientific community and the private population expressed curiosity about this rare and unique event; hence, the researcher Dionys Hellin generated an equation in 1895 that could predict the occurrence of multiple births, which was termed the “Hellin-Zeleny rule” [5].

According to statistics based on American inhabitants published in 2018 and 2019 by the Centers for Disease Control and Prevention, the prevalence of multiple pregnancies triplet or larger gestations peaked in 1998, with a total of 193.5 cases per 100,000 births. However, an imminent declining pattern is currently observed; in 2016, statisticians indicated that they had found the lowest level reported in two decades, a total of 101.4 cases per 100,0000 births [6]. In addition, when observing the data obtained from the population analyzed in 2018, they were again faced with the lowest relationship, with a total of 93.0 cases per 100,000 births [6].

High-order fetal gestation creates a high risk of morbidity and mortality for both mother and fetuses [7, 8]. Numerous complications have been reported in these versus spontaneous pregnancies, predominantly chromosomal alterations, antepartum and postpartum hemorrhages, hypertensive disorders in early onset pregnancy, premature rupture of membranes, polyhydramnios, perinatal mortality, gestational diabetes mellitus, anemia, preeclampsia, and preterm births accompanied by low birth weight [7–9].

In the literature, complications have been determined as well as evidence that adequate multidisciplinary management accompanied by new technologies prior to pregnancy and quality maternal nutrition directly impact success in the gestational and neonatal periods [4, 10]. In Honduras, there are no reports of quadruple pregnancies as a result of ART with artificial insemination, such as the one described in this work. This study aimed to report a case of quadri-chorionic quadri-amniotic pregnancy, which, due to its course without complications, reaches a full-term pregnancy, maternal personal history, the risk of the process, adequate birth weights, and the favorable results obtained, become vital and relevant within the obstetrics field, particularly ART.

## Case presentation

A 33-year-old married white woman from a rural area with a history of suspected endometriomas on serial ultrasound underwent an ovarian function study in 2012. The patient and her partner have a medium–high educational and economic status. At that time, a contrast radiography of the uterine tubes and uterus (hysterosalpingography) revealed bilateral obstruction of the proximal fallopian tubes. Laparoscopic surgery was performed one year later, and findings included endometriosis stage 2 with greater involvement of the left tube. The foci of the endometriosis were cauterized, and drug treatment (leuprorelin acetate 3.75 mg IM) every 28 days for 4 months was used to eradicate the endometriotic foci and improve the tube peritoneal function. She sought care at a private clinic to start treatment and achieve a pregnancy due to a 2-year history of infertility secondary to bilateral tubal affection. In 2014, pharmacological treatment began, and four failed attempts were registered during four months of treatment with clomiphene citrate (100–200 mg/day for 5 days) and letrozole (5.0–7.5 mg/day for 5 days). Programmed intercourse achieving pregnancy but with consequent chemical abortion. Artificial insemination employed in 2015 using follitropin alfa 75 IU (5.5 μg Gonal-F) and choriogonadotropin alfa 250 μg/0.5 mL (Ovidrel) achieves 5 follicles in the left ovary and one mature ovum in the right ovary. One month later, a semen sample was trained with the swim up technique and the first successful pregnancy was achieved with normal prenatal controls. Prenatal controls mainly comprise a complete medical history, periodic clinical examinations, tetanus vaccination, perinatal risk assessment, gynecological-obstetric examination, delivery plan, etc. The delivery was made by caesarean section for pelvic presentation and a female newborn was delivered without perinatal complications.

In 2018, the patient returned with the aim of conceiving again. Hysterosalpingography and hormonal profiles were within normal parameters. Low-complexity oral pharmacological treatment was started using programmed intercourse plus the implementation of clomiphene (100–200 mg/day for 5 days), and three cycles were applied for 3 months. Despite reaching a dosage of clomiphene citrate 200 mg, only an ovarian follicle was obtained.

The patient was consulted, and because of the success achieved three years previously, it was decided to proceed again with artificial insemination. Ovarian stimulation was started on January 18, 2018, with 150 IU follitropin alfa (11.0 μg Gonal-F). The dose was doubled compared to the first pregnancy due to the patient’s older age and the lack of development with clomiphene and letrozole. Using this approach, three follicles on the right and five follicles on the left of acceptable size were achieved and the dose of follitropin alfa was decreased to 75 IU. On January 28, the patient had four 16-mm ovarian follicles on each side; two days later, a dominant 24 mm follicle accompanied by two 18 mm follicles were noted on the left side in addition to two 18-mm follicles on the right side. The five follicles were mature and of suitable condition for ovulation; therefore, a single dose of choriogonadotropin alfa 250 μg/0.5 mL (Ovidrel) was indicated.

Artificial insemination was performed on January 30, a good-quality sperm sample was obtained from the couple and was trained again by the swim-up technique. The insemination proceeded without complications: a fine cannula was inserted under ultrasound guidance into the uterine cervix 5 mm from the uterine fundus, a sperm sample was injected, and the patient rested for one hour. Luteal support with 200 mg of micronized progesterone daily for 13 weeks was prescribed. At three weeks post-insemination, the patient underwent qualitative β-Hcg confirmation of the successful pregnancy by insemination.

**Fig. 1:**
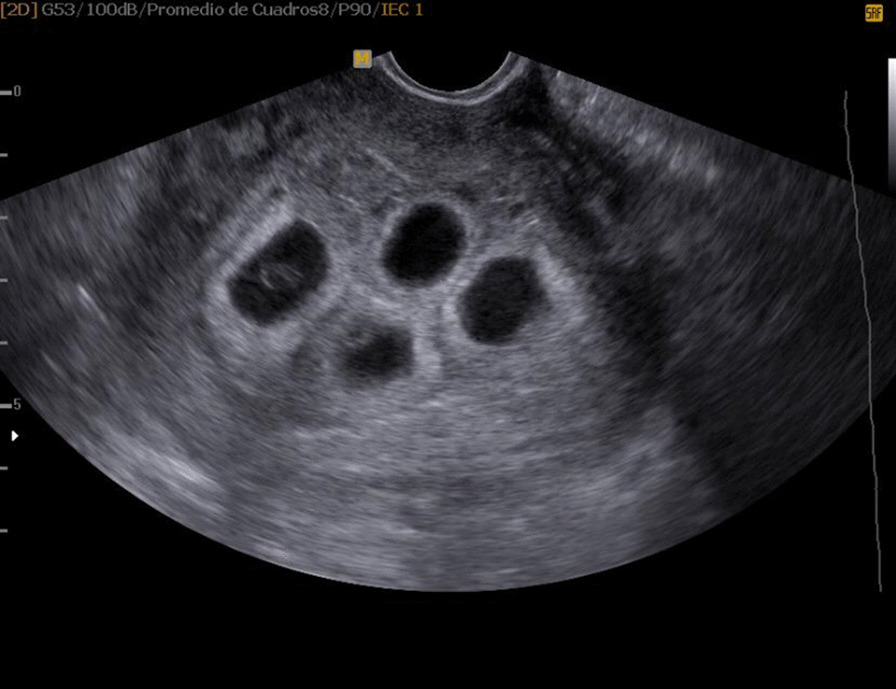
Clear appreciation of the four independent gestational sacs. The yolk sacs of the two on the left side are visible. Good chorionic reactions are visible

**Fig. 2:**
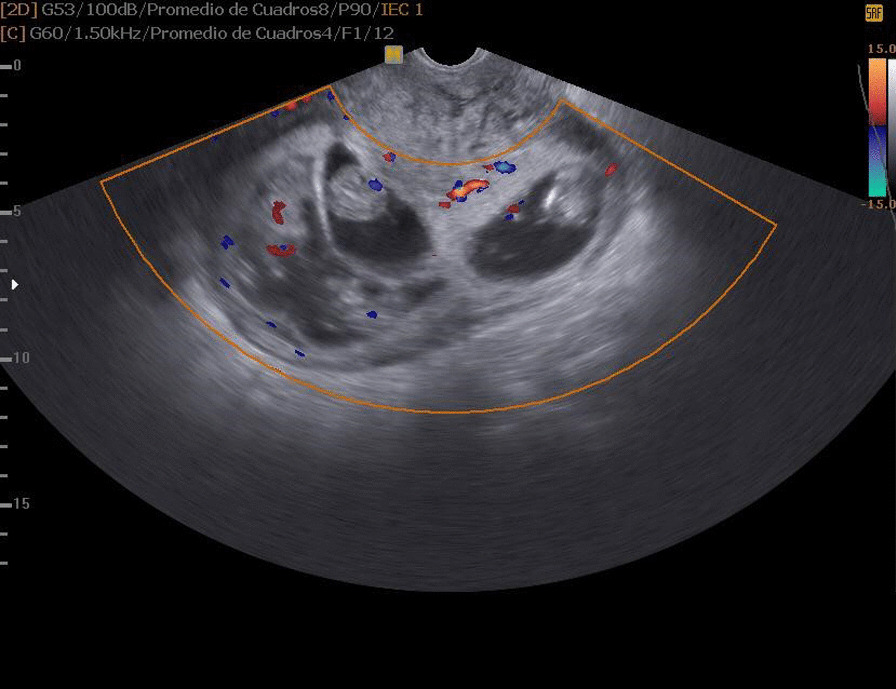
Gestational sacs and their embryos on Doppler ultrasound

At the sixth week of pregnancy, the beta-human chorionic gonadotropin (β-hCG) level was 137,000 mIU/mL (suggestive of multiple pregnancy), and an obstetric ultrasound revealed quadri-amniotic gestation with 3–5 mm yolk sacs with good chorionic reaction, located in the uterine fundus. No areas of detachment or decidual hemorrhage were observed, and the measurements corresponded to a 22 mm sacs and 3 mm embryos at six weeks and five days of gestation with a probable due date of October 24, 2019 (Figs. 1 and 2). The pregnancy progressed without relevant fetal or maternal complications, maternal anemia, urinary tract infections, respiratory infections, fetal chromosomal disorders, maternal obesity, pre-eclampsia, edema, or uterine hypotonia. Quality maternal nutrition, optimal physical conditioning, and patient age have considerable influence.

Morphological ultrasounds performed at gestational weeks 13 and 24 by a perinatologist revealed normal organ development without evidence of anatomical alterations in any of the fetuses. At 30 weeks, lung maturation inducers betamethasone 12 mg IM for two days were prescribed. The patient attended a total of 13 prenatal check-ups, monthly until week 24, every 15 days until week 31, and then weekly until the end of the pregnancy. The last patient check-up was at 36.3 gestation weeks (GW), presenting a blood pressure of 130/85 mmHg, slightly elevated compared to maternal parameters in previous evaluations. An obstetric ultrasound involved significant difficulty differentiating between the fetal heartbeats. The patient reported feeling discomfort, dyspnea, inability to fall asleep, walking difficulties, and excessive weight gain. Thus, delivery was scheduled.

The patient underwent surgery 1 week later on October 5 upon reaching 37.2 GW, with a weight of 206 lb between her and the fetuses (154 lb initial maternal weight). She was transferred to a private clinic for maternal and fetal well-being and the care and monitoring of the future newborns.

**Fig. 3:**
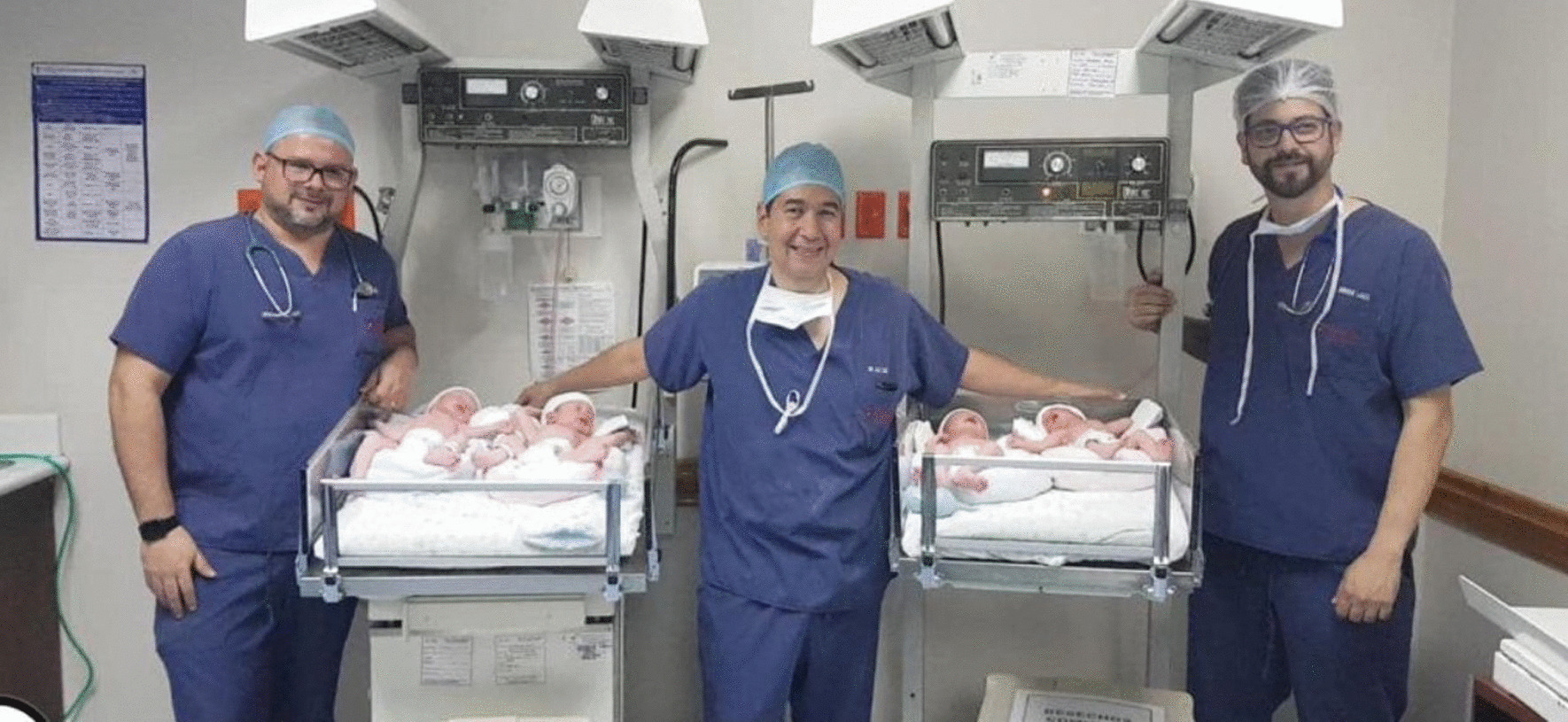
Clinical care team with the newborn babies

Cesarean section was performed without maternal or fetal complications, accompanied by a multidisciplinary team of specialists (two gynecologists, two pediatricians, neonatologists, anesthesiologists, and nursing staff) and incubators prepared to avoid or substantially minimize perinatal complications. Four children were born, two girls and two boys, with normal APGAR scores and good fetal status, weighing: 2600 g, 2740 g, 2380 g, and 1700 g. The newborns did not require ventilatory support, oxygen therapy, antibiotics, or any prophylactic intervention, and no expected perinatal complications occurred. A multidisciplinary team was ready to resolve the possible eventuality of uterine atony, but no intervention was necessary (Fig. 3). The uterus acquired hypertonia physiologically after evacuation. The patient was discharged on the second postpartum day with the four newborns, during which time she presented ataxic gait complications, making it difficult to maintain her balance without the weight of the pregnancy, but this resolved completely after 15 days.

**Fig. 4:**
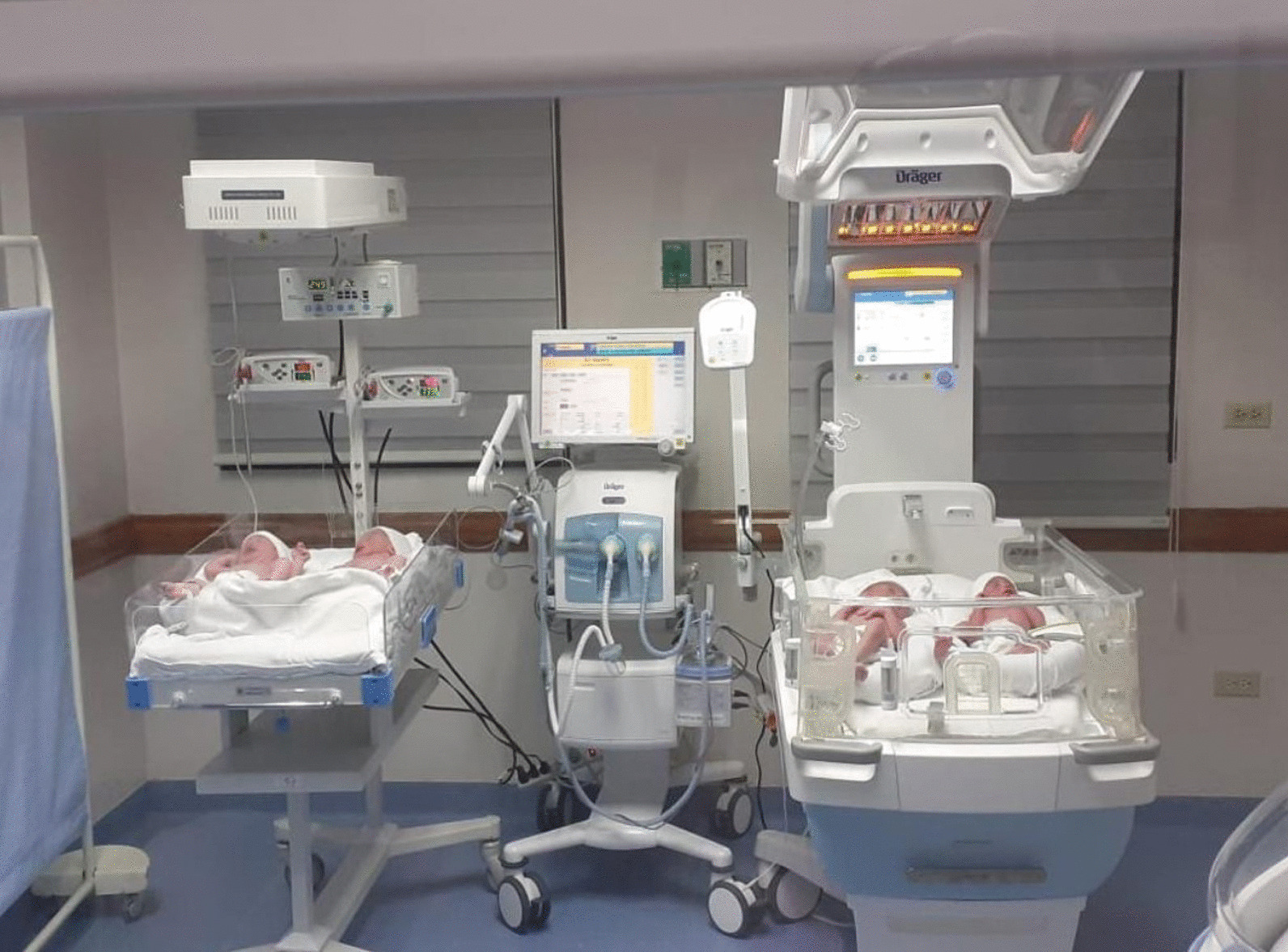
The neonates within a few hours after birth

The four children are currently in good health and have shown no alterations in subsequent evaluations (Fig. 4). This case represents an achievement in the obstetric field and in the development of ART given the difficulty and risk posed by this type of multiple pregnancy and the arduous task of achieving a successful full-term multiple pregnancy. The patient did not want to be sterilized, but agreed to underwent tubal ligation during delivery.

## Discussion

High-order pregnancies have become a challenge for ART centers given the complexity of their management and strict control that must be followed to prevent complications that can be expected in these cases [11, 12].

Once pregnancy is established, the couple faces the dilemma of a high-order pregnancy and an attempt is made to continue with all fetuses, since the couple usually does not accept total interruption of the pregnancy, especially when they have a history of infertility [13]. In Honduras, the law does not allow fetal interruption or reduction, a process that would improve the outcomes of these pregnancies [14], which leads to the association of inherent problems that will intervene in long-term survival and morbidity [5].

In the case presented, total success was achieved in every sense due to the mother's excellent physical condition, and her attachment to the pregnancy which contributed to her perseverance in following the treatment indicated by her doctor. In gynecological-obstetric terms, this case represents an achievement in the clinical practice of ART not previously described in the country, with excellent maternal and perinatal results.

In newborns, there is an increased risk of prematurity (26–35 GW), low birth weight (mean 1046–1778 g, 14), cerebral palsy, death [15], and mortality (20 times higher in the first month of life, [11, 12, 16–18]). The data presented in this case contrast the poor prognosis usually expected in this type of pregnancy; the mother and fetuses did not present any type of complication; the mother had a controlled pregnancy with blood pressures, ultrasound findings, and laboratory tests all within normal parameters and the cesarean section not showing alterations and being scheduled at term.

Preterm birth is the most common maternal complication in high-order pregnancies, of which 90% of infants are born prematurely [19], with the average gestational age at delivery for quadruplets being at approximately 29 weeks and 5 days [20]. The importance of this case lies in the success achieved in bringing the pregnancy to term at 37.2 GW, exceeding the usual range observed in quadruple pregnancies [21]. In 2016, the United States reported that 93% of quadruplet pregnancies were delivered before 34 weeks of gestation [6].

In this study, the fetal weights were adequate (2600 g, 2740 g, 2380 g, and 1700 g) with better weighing than in other case series of high-order fetal pregnancies [21], It has been reported that 77.1% of quadruple pregnancy birth weights are below 1500 g and 96.2% are below 2500 g, and a limited nutrient supply could be responsible for low birth weight [22]. The newborns were discharged together with the mother without early or late respiratory complications, did not present hyperbilirubinemia or other complications, and achieved the objective of all reproduction techniques: a healthy baby at home.

The patient attended 13 prenatal check-ups and was evaluated every week in recent months. There is no consensus about the periodicity of prenatal controls in high-order gestations, having a stricter and more exhaustive control in the last few weeks improves the maternal–fetal prognosis, avoids complications, and controls the weight of each fetus to enable the identification of discrepancies or alterations of the amniotic fluid [21, 23]. It should be noted that the patient came from a rural area, where she had limited access to specialized health services, and it was necessary to transfer her to an urban area in the final few months of the pregnancy because of the possibility of premature rupture of membranes or pre-eclampsia that would accelerate the evacuation of the pregnancy.

In high-order pregnancies, the literature reports scheduled evacuations before term; in Honduras, neonatal units usually do not have the best resources to guarantee fetal well-being and decrease morbidity rates; therefore, the pregnancy was allowed to evolve until term. In this case, the well-being and comfort of the mother had great influence, as her quality of life was not affected by the quadruple pregnancy. The authors suggest that clinicians not intervene if a pregnancy continues without eventualities and allow its natural evolution as long as the fetal and maternal well-being tests provide adequate results.

The management of a high-order pregnancy is challenging for obstetricians in charge of the well-being of pregnant women and their fetuses. A key point is the close monitoring of maternal well-being, which could be the key to the full development of a successful pregnancy. The case presented here represents an achievement in every way.

## Conclusion

In Honduras, there are currently no statistical reference data for determining the annual incidence of high-order pregnancies, even less than those caused by ART. The present work represents one of the first reports in the field of artificial insemination in country with successful quadruple pregnancy resolution. The international literature on high-order pregnancies remains limited, because of its low incidence; this lack of concrete data makes it difficult to choose a treatment to avoid complications.

This paper presented the parameters of prenatal care, appropriate management approach, and successful resolution without maternal–fetal complications despite the inherent risks of this type of pregnancy. For achieving and completing successful pregnancy other factors were also associated, as family support, the mother's attachment to the pregnancy and the patient's good nutrition through a strict diet.

## Data Availability

Patient’s files and datasets used to support the findings of this study are restricted to protect the privacy of clinical data. Data are available to investigators who comply the criteria for access to confidential data under request. Requests for access to these data should be directed to César Alas-Pineda: cesar_alas10@hotmail.com.

## Abbreviations

ART: Assisted reproductive technologies
GW: Gestation weeks
